# A Systematic Umbrella Review on the Epidemiology of Modifiable Health Influencing Factors and on Health Promoting Interventions Among University Students

**DOI:** 10.3389/fpubh.2020.00137

**Published:** 2020-04-28

**Authors:** Pavel Dietz, Jennifer L. Reichel, Dennis Edelmann, Antonia M. Werner, Ana Nanette Tibubos, Markus Schäfer, Perikles Simon, Stephan Letzel, Daniel Pfirrmann

**Affiliations:** ^1^Institute of Occupational, Social and Environmental Medicine, University Medical Centre of the University of Mainz, Mainz, Germany; ^2^Department Sport Medicine, Rehabilitation and Disease Prevention, Institute of Sport Science, Johannes Gutenberg University, Mainz, Germany; ^3^Department of Psychosomatic Medicine and Psychotherapy, University Medical Center of the Johannes Gutenberg University Mainz, Mainz, Germany; ^4^Department of Communication, Johannes Gutenberg University, Mainz, Germany

**Keywords:** university students, modifiable health influencing factors, epidemiology, intervention, health promotion

## Abstract

**Background:** Universities represent an important setting for health promotion. The unique collective of university students is of particular relevance since they are the leaders, decision-makers, and parents of tomorrow. In this context, modifiable health influencing factors as well as interventions to prevent these, play a crucial role. Therefore, the present umbrella review aims to (i) provide an overview of review articles addressing epidemiological issues (prevalence and determinants) of modifiable health influencing factors in university students and (ii) to provide an overview of review articles addressing the evidence of interventions to promote/enhance modifiable health influencing factors in university students.

**Methods:** A systematic literature search was performed in the databases PubMed, Cochrane Reviews Library und Web of Science according to the PRISMA guidelines. Only systematic reviews and meta-analyses were included. The AMSTAR-2-Tool was used for the quality assessment.

**Result:** The initial search resulted in 10,726 records of which 81 fulfilled the inclusion criteria, with a further distinction in articles with an epidemiological focus (*n* = 39) and in articles with interventional approaches (*n* = 42). Topics of the different review articles ranged from physical activity over mental health, substance use, sleep, diet and nutrition, and media consumption. Many review articles had a specific focus on medical and nursing students and originated from the U.S.A., U.K., or China.

**Discussion:** This umbrella review provides an overview of review articles on the epidemiology of modifiable health influencing factors and on the evidence of interventions targeting these factors among university students. Thereby, experts as well as stakeholders in the field could gain insights into crucial target points for health promotion. It identifies research gaps in terms of study region and groups of students.

## Introduction

According to the World Health Organization (WHO) health is more than just the current condition of a person, but rather “a resource for everyday life” (1). It is created and lived by people within the settings of their everyday life: where they learn, work, play, and love (1), emphasizing the interconnectedness between individuals and their environments. In 2015, an international expert group formulated the Okanagan Charter and pointed out that universities are an important setting of everyday life for health promotion (2). They further stated that from a public health point of view, the unique collective of university students would be of particular relevance (2) since they are the leaders, decision-makers, and parents of tomorrow. Therefore, health promotion in students could be sustainable and beneficial for the general society. Additionally, the students' entrance into a new living environment, called university, causes changes in the home environment, work environment, and recreational environment (3). Furthermore, in the critical period of young adulthood (18–25 years), students are potentially vulnerable for risky health behavior such as drinking or physical inactivity (4).

In the context of health promotion in university students, modifiable health influencing factors play a crucial role. These factors encompass, for instance, physical activity, nutrition, substance, and media use (5). What they all have in a common is that they can be modified instantly and may have immediate or long-term effects on an individual's health (6). In contrast, other relevant health influencing factors like age, gender, or genetics cannot be changed instantly. Given the fact that the time being enrolled at a university as a student is relatively short (regularly 3–4 years for bachelors and 2 years additional years for masters), modifiable health influencing factors play a significant role for health promotion among university students. Taking the great potential of these factors for health promotion and prevention into account, it is important to provide an evidence base on (i) the epidemiology of modifiable health influencing factors (prevalence and determinants) and (ii) interventions to promote/enhance modifiable health influencing factors in university students. On the one hand, information on the epidemiology will be relevant to identify these factors and potential risk groups among university students, which might be of particular interest for health promotion. On the other hand, the information will be important in order to identify potential scientific knowledge gaps regarding specific health factors, student collectives or countries. In addition, from a public health point of view, knowledge regarding interventions to promote modifiable health influencing factors will be of significant relevance to develop and implement evidence-based student health interventions in a more personalized way and tailored to specific risk groups.

Currently, hundreds of review articles regarding the epidemiology of modifiable health influencing factors of university students and according interventions can be found in the literature. To name a few, Keating et al. focused on physical activity behavior in college students (7), Castro et al. on sedentary behavior in university students (8), Elani et al. on stress among dental students (9), McKenna et al. on psychological wellbeing of international students in the health professions (10), Bennett et al. on smoking behavior of college students (11), Davoren et al. on alcohol consumption of university students in Ireland and the UK (12), Candido et al. on drug consumption of medical students (13), or Cassidy et al. on sexual behavior (14) of university students. Most of these reviews have in common, that they only address one specific modifiable health influencing factor in either one specific collective of students (e.g., dental students, medical students, college students), or in university students per se, in a specific region or worldwide. The large amount of studies in this field, however, makes it difficult to gain an overview about existing literature, to generate a synthesis of the evidence as well as to identify a potential lack of knowledge or research gaps (e.g., regarding rarely explored modifiable health influencing factors, specific student collectives or regions) respectively. Therefore, the present umbrella review aims to (i) provide an overview of review articles addressing epidemiological issues (prevalence and determinants) of modifiable health influencing factors in university students, (ii) to provide an overview of review articles addressing the evidence of interventions to promote/enhance modifiable health influencing factors in university students, enabling us to, (iii) detect potential health-related risk groups in the student population regarding, for example, field of study or region, and to (iv) identify health-related knowledge gaps in the student population, for example, regarding field of study or region.

## Materials and Methods

The decision to perform an umbrella review was based on the large amount of single studies and review articles dealing with health of university students. The present review was performed according to the “Preferred Reporting Items for Systematic Reviews and Meta-Analyses” (PRISMA) Guidelines (15).

### Search Strategy

A systematic literature search was carried out in the electronic databases PubMed, Cochrane Reviews Library, and Web of Science. For the data base PubMed, the following three-level search term (collective, institution, and topic) was created using Boolean operators: (student OR students) AND (university OR college OR “higher education” OR academy OR “tertiary education” OR school) AND (health^*^ OR wellbeing OR disease OR disorder OR illness OR sickness OR “physical activ^*^” OR “physical inactiv^*^” OR exercise OR fitness OR sedentary OR sedentariness OR nutrition OR diet OR “substance use” OR “substance abuse” OR “substance consumption” OR “substance misuse” OR “drug use” OR “drug abuse” OR “drug consumption” OR “drug misuse” OR doping OR “pharmacological neuroenhancement” OR “pharmacological cognitive enhancement” OR alcohol OR smoking OR tobacco OR marijuana OR cannabis OR addiction OR “media use” OR “media consumption” OR “media usage” OR “internet use” OR “internet consumption” OR “mobile phone use” OR “mobile phone consumption” OR “smart phone use” OR “smart phone consumption” OR “cell phone use” OR “cell phone consumption” OR stress OR anxiety OR mobbing OR bullying OR mindfulness OR satisfaction OR “quality of life” OR self-concept OR “risk behavio^*^” OR “risk attitude” OR resilience OR vaccination OR vaccines OR “hand-wash^*^” OR “sexual behavio^*^” OR “sun protection” OR “sun burn”). Since we searched for [All Fields] in PubMed, MeSH terms were generated automatically. For the other databases, this search term was adapted following the individual Cochrane Reviews Library and Web of Science search guidelines. If possible, limits for article type (review) and language (German and English) were activated. No time limits were set. The search was completed on February 28th, 2019.

### Inclusion Criteria

In this systematic umbrella review, all review articles had to fulfill the following inclusion criteria: (1) being a systematic review or meta-analysis. To be classified as systematic, at least points 6 to 9 of the PRISMA checklist had to be fulfilled; (2) focusing exclusively on students from universities, colleges and universities of applied sciences. Studies investigating mixed student collectives (e.g., pupils, medical residents) were excluded (3) addressing the prevalence or/and determinants of at least one modifiable health influencing factor; or/and (4) addressing the effects of at least one intervention to promote at least one modifiable health influencing factor; (5) being published in a peer-reviewed journal in (6) English or German language. All kinds of study designs (observational, cross-sectional, longitudinal, randomized, non-randomized, and uncontrolled) were included.

### Selection Process and Data Extraction

**Figure 1:**
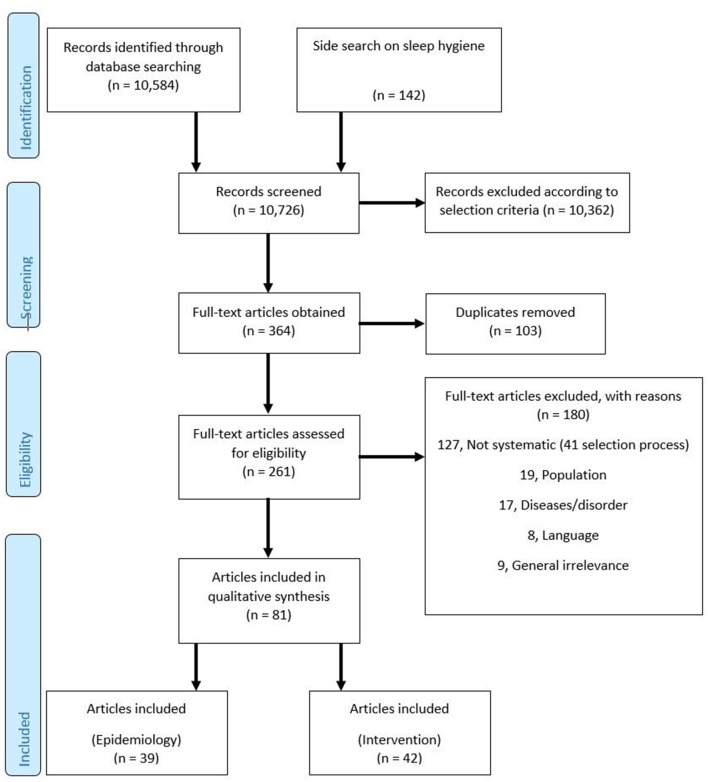
PRISMA flow diagram.

The flow chart in Figure 1 provides a transparent documentation of article elimination. Two reviewers independently screened title and abstract of all potentially relevant articles. Then, two independent reviewers evaluated full texts and removed duplicates. Specific reasons for exclusion are presented in the flow chart. Uncertainties were discussed in the researcher team in order to achieve consensus. The data extraction was also performed according to the dual control principle. Relevant data of the included articles were summarized in tables and checked for accuracy by another researcher. Uncertainties were discussed in the reviewer team in order to achieve consensus.

The following data were extracted: (1) authors; (2) year of review; (3) number of included single studies (4) subject characteristics (e.g., field of study, age, gender, and race) (5) data on modifiable health influencing factors (6) important findings were highlighted (7) result of the quality assessment (if carried out). Furthermore, the number of participants per review article was calculated. Based on the descriptive distribution of the number of original articles per country in each included review article, a color scheme was created to graphically depict countries with high scientific output on modifiable health influencing factors among students and countries with less scientific output. If the study countries of the original article were not provided in the included articles (e.g., only information about the continent), the study country was extracted from the original article.

### Quality Assessment

For all studies with scope on interventions, an additional quality assessment was performed using the “assessing the methodological quality of systematic reviews (AMSTAR) 2” tool (16). The AMSTAR 2 contains 16 items for a critical appraisal of systematic reviews. It rates the overall confidence of a review article in four categories (from high to critically low) through spotting critical and non-critical weaknesses. Therefore, the results of the quality assessment should not be used to obtain an overall score, but to identify critical domains (15).

## Results

The initial search resulted in 10,726 records through database searching (*n* = 7,178 from PubMed; *n* = 3,378 from Web of Science; *n* = 28 from Cochrane Library), and from an additional side search on sleep hygiene among students (*n* = 142). Of these, 10,362 records were excluded after title and abstract screening according to the selection criteria, resulting in a number of 364 potentially appropriate articles. After removal of duplicates, 261 full texts were available for detailed assessment. One hundred and eighty articles were excluded for specific reasons, see flow chart (Figure 1). Thus, 81 articles, comprising 2,703 original articles, met the eligibility criteria and were included in this umbrella review. They were further distinguished in articles with an epidemiological focus (*n* = 39; comprising 1,525 original articles) and in articles with interventional approaches (*n* = 42; comprising 1,178 original articles).

**Figure 2:**
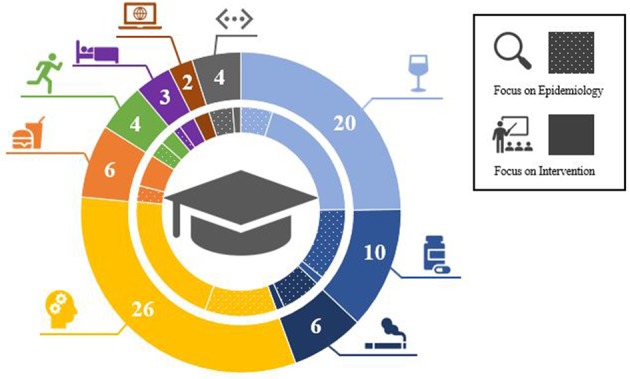
Number of included review articles sorted by topic and whether they have focus on epidemiology or intervention aspects.

Table 1 provides a summary of the main characteristics of the identified articles, including: collective, region, number of original articles included, and quality assessment. The identified articles (*n* = 81) were published between 2007 and 2018 and fall into one of seven broad categories, namely “substance use” (*n* = 36), “mental health / wellbeing” (*n* = 26), “diet and nutrition” (*n* = 6), “physical activity” (*n* = 4), “sleep hygiene” (*n* = 3), “media consumption” (*n* = 2), “others” (*n* = 4); Figure 2. The different categories display the variety of health topics throughout the review articles. The quality assessment for the interventional articles indicated mainly a critically low (*n* = 23) and low (*n* = 13) quality, demonstrating a potential risk for bias and only few articles were identified with moderate quality (*n* = 6). The investigated collective was not further classified in most of the review articles, with only a few exceptions (e.g., nursing students, medical students, or dental students). Mainly university/college students as a general population were under investigation.

**Table 1:** Main characteristics of the articles included in the systematic umbrella review.

**References**	**Title**	**Region**	**Collective**	**Categories assessed**	**N articles**	**QA**
**Epidemiological SR/MA**						
Aresi et al. (17)	Drinking, drug use, and related consequences among university students completing study abroad experiences: A systematic review	Mainly USA	College students	Substance use–alcohol, drugs	S18	
Bavarian et al. (18)	The illicit use of prescription stimulants on college campuses: A theory-guided systematic review	-	College students	Substance use–drugs	S62	
Bennett et al. (11)	College anti-smoking policies and student smoking behavior: A review of the literature	USA	College students	Substance use–smoking	S11	
Bennett and Holloway (19)	Motives for illicit prescription drug use among university students: A systematic review and meta-analysis	International	University students	Substance use–drugs	M29	
Benson et al. (20)	Misuse of stimulant medication among college students: A comprehensive review and meta-analysis	-	Undergraduate students	Substance use–drugs	S30/M20	
Blavos et al. (21)	Marijuana and college students: A critical review of the literature	USA	Undergraduate students	Substance use–drugs	S35	
Bruening et al. (22)	The struggle is real: A systematic review of food insecurity on postsecondary education campuses	International	Postsecondary students	Diet and nutrition	S59	
Brunsting et al. (23)	Predictors of undergraduate international student psychosocial adjustment to US universities: A systematic review from 2009-2018	USA	Undergraduate students	Mental Health/Wellbeing	S30	
Candido et al. (13)	The use of drugs and medical students: A literature review	Brazil	Medical students	Substance use–drugs	S16	
Castro et al. (8)	Correlates of sedentary behavior in university students: A systematic review	International	University students	Physical health	S129	
Cheney et al. (24)	Smoking and membership in a fraternity or sorority: A systematic review of the literature	USA	University students	Substance use–smoking	S19	
Davoren et al. (12)	Alcohol consumption among university students in Ireland and the United Kingdom from 2002 to 2014: A systematic review	Ireland; UK	University students	Substance use–alcohol	S29	
Elani et al. (9)	A systematic review of stress in dental students	International	Dental students	Mental Health/Wellbeing	S124/M21	
Elliott et al. (25)	Does family history of alcohol problems influence college and university drinking or substance use? A meta-analytical review	Mainly USA	College students	Substance use–alcohol, drugs	S65	
Fedewa et al. (26)	Change in weight and adiposity in college students: A systematic review and meta-analysis	-	College students	Physical health	M49	
Fevrier et al. (27)	Policy implications and research recommendations: A review of Hookah use among US college students	USA	College students	Substance use–smoking	S115	
Finger et al. (28)	Use of methylphenidate among medical students: A systematic review	-	Medical students	Substance use–drugs	9	
Gambla et al. (29)	College tanning behaviors, attitudes, beliefs, and intentions: A systematic review of the literature	USA	College students	Others	S23	
Gebrie et al. (30)	Prevalence and predictors of khat chewing among Ethiopian university students: A systematic review and meta-analysis	Ethiopia	University students	Substance use–drug	S24/M24	
Guerra et al. (31)	Tobacco consumption among college students: A systematic review	International	University students	Substance use–smoking	S62	
Haghdoost and Moosazadeh (32)	The prevalence of cigarette smoking among students of Iran's universities: A systematic review and meta-analysis	Iran	University students	Substance use–smoking	M22	
Haidar et al. (33)	Stress, anxiety, and weight gain among university and college students: A systematic review	International	University students	Mental Health/Wellbeing	S25	
Hurst et al. (34)	College student stressors: A review of the qualitative research	International	College students	Mental Health/Wellbeing	S40	
Jahrami et al. (35)	Eating disorders risk among medical students: a global systematic review and meta-analysis	International	Medical students	Diet and nutrition	S18/M18	
Karam et al. (3)	Alcohol use among college students: An international perspective	International	College students	Substance use–alcohol	S26	
Labrague et al. (36)	Examining stress perceptions and coping strategies among Saudi nursing students: A systematic review	Saudi Arabia	Nursing students	Mental Health/Wellbeing	S11	
Li et al. (37)	Prevalence of sleep disturbances in Chinese university students: A comprehensive meta-analysis	China	University students	Sleep	M76	
McGowan and Murray (38)	Exploring resilience in nursing and midwifery students: a literature review	Mainly USA	Nursing students	Mental Health/Wellbeing	S8/M0	
Mortier et al. (39)	The prevalence of suicidal thoughts and behaviors among college students: A meta-analysis	International	College students	Mental Health/Wellbeing	S66/M36	
Nahar et al. (40)	Skin cancer knowledge, attitudes, beliefs, and prevention practices among medical students: A systematic search and literature review	International	Medical students	Others	S21	
Newman et al. (41)	Estimate of undergraduate university student alcohol use in China: A systematic review and meta-analysis	China	Undergraduate students	Substance use–alcohol	M30	
Papazisis et al. (42)	Prevalence of cannabis use among medical students: A systematic review and meta-analysis	International	Medical students	Substance use–drugs	S38	
Roncero et al. (43)	Substance use among medical students: A Literature Review 1988-2013	International	Medical students	Substance use–drugs	S106	
Sasso et al. (44)	Moral distress in undergraduate nursing students: A systematic review	International	Nursing students	Mental Health/Wellbeing	S4	
Schry and White (45)	Understanding the relationship between social anxiety and alcohol use in college students: A meta-analysis	-	College students	Substance use–alcohol	S44	
Shao et al. (46)	Internet addiction detection rate among college students in the people's Republic of China: A meta-analysis	China	College students	Media consumption	M26	
Stellefson et al. (47)	eHealth literacy among college students: A systematic review with implications for eHealth education	Mainly USA	College students	Others	S7	
Thomas and Revell (48)	Resilience in nursing students: An integrative review	International	Nursing students	Mental Health/Wellbeing	S9	
Zhang et al. (49)	Prevalence of internet addiction in medical students: A meta-analysis	International	Medical students	Media consumption	M10	
**Interventional SR/MA**						
Akinla et al. (50)	A systematic review of the literature describing the outcomes of near-peer mentoring programs for first year medical students	International	Medical students	Others	S5/M0	Critically low
Alzahem et al. (51)	Stress management in dental students: A systematic review	International	Dental students	Mental Health/Wellbeing	S7	Critically low
Appiah-Brempong et al. (52)	Motivational interviewing interventions and alcohol abuse among college students: A systematic review	USA	College students	Substance use–alcohol	S13	Low
Berman et al. (53)	Mobile interventions targeting risky drinking among university students: A review	International	University students	Substance use–alcohol	S7	Low
Bhochhibhoya et al. (54)	The use of the internet for prevention of binge drinking among the college population: A systematic review of evidence	Mainly USA	College students	Substance use–alcohol	S14	Low
Bonthuys and Botha (55)	Tomatis® Method comparative efficacy in promoting self-regulation in tertiary students: A systematic review	International	College students	Mental Health/Wellbeing	S35/M0	Low
Carey et al. (56)	Individual-Level Interventions to reduce college student drinking: A meta-analytic review	Mainly USA	College students	Substance use–alcohol	M62	Critically low
Carey et al. (57)	Computer-delivered interventions to reduce college student drinking: A meta-analysis	International	College students	Substance use–alcohol	M35	Critically low
Carey et al. (58)	Face-to-Face vs. computer-delivered alcohol interventions for college drinkers: A meta-analytic review, 1998 to 2010	Mainly USA	College students	Substance use–alcohol	M48	Critically low
Carey et al. (59)	Alcohol interventions for mandated college students: A meta-analytic review	USA	College students	Substance use–alcohol	M30	Critically low
Christoph and An (60)	Effect of nutrition labels on dietary quality among college students: A systematic review and meta-analysis	International	College students	Diet and nutrition	S22/M10	Critically low
Conley et al. (61)	A meta-analysis of universal mental health prevention programs for higher education students	Mainly USA	Higher education students	Mental Health/Wellbeing	S103/M90	Critically low
Conley et al. (62)	A meta-analysis of the impact of universal and indicated preventive technology-delivered interventions for higher education students	Mainly USA	Higher education students	Mental Health/Wellbeing	S48/M41	Critically low
Deliens et al. (63)	Dietary interventions among university students: A systematic review	International	University students	Diet and nutrition	S20	Low
Dietrich et al. (64)	Effectiveness of sleep education programs to improve sleep hygiene and/or sleep quality in college students: A systematic review	USA	College students	Sleep	S4	Low
Dotson et al. (65)	Stand-Alone personalized normative feedback for college student drinkers: A meta-analytic review, 2004 to 2014	Mainly USA	College students	Substance use–alcohol	S8	Moderate
Foxcroft et al. (66)	Social norms information for alcohol misuse in university and college students (Review)	International	College students	Substance use–alcohol	S70/M63	Moderate
Friedrich and Schlarb (67)	Let's talk about sleep: A systematic review of psychological interventions to improve sleep in college students	International	College students	Sleep	S27/M27	Moderate
Galbraith and Brown (68)	Assessing intervention effectiveness for reducing stress in student nurses: Quantitative systematic review	International	Nursing students	Mental Health/Wellbeing	S16	Critically low
Gulliver et al. (69)	Technology-based interventions for tobacco and other drug use in university and college students: A systematic review and meta-analysis	International	University students	Substance use–smoking, drugs	S12/M6	Moderate
Ickes et al. (70)	Alcohol abuse prevention programs in college students	USA	College students	Substance use–alcohol	S49	Critically low
Kelly et al. (71)	Systematic review of dietary interventions with college students: Directions for future research and practice	-	College students	Diet and nutrition	S14	Critically low
Labrague et al. (72)	A literature review on stress and coping strategies in nursing students	International	Nursing students	Mental Health/Wellbeing	S13	Critically low
Labrague et al. (73)	An integrative review on coping skills in nursing students: Implications for policymaking	International	Nursing students	Mental Health/Wellbeing	S27	Low
Li et al. (74)	Interventions to promote mental health in nursing students: A systematic review and meta-analysis of randomized controlled trials	International	Nursing students	Mental Health/Wellbeing	S12/M12	Moderate
Lo et al. (75)	Group interventions to promote mental health in health professional education: A systematic review and meta-analysis of randomized controlled trials	International	Health professional students	Mental Health/Wellbeing	S24/M19	Low
Lupton and Townsend (76)	A systematic review and meta-analysis of the acceptability and effectiveness of university smoke-free policies	Mainly USA	University students	Substance use–smoking	S19/M12	Critically low
Maselli et al. (77)	Promoting physical activity among university students: A systematic review of controlled trials	International	University students	Physical health	S28	Low
McCarthy et al. (78)	Nursing and midwifery students' stress and coping during their undergraduate education programmes: An integrative review	International	Nursing students	Mental Health/Wellbeing	S25	Critically low
McConville et al. (79)	Mindfulness training for health profession students–the effect of mindfulness training on psychological well-being, learning and clinical performance of health professional students–A systematic review of randomized and non-randomized controlled trials	-	Health professional students	Mental Health/Wellbeing	S19	Low
Moreira et al. (80)	Social norms interventions to reduce alcohol misuse in university or college students (Review)	Mainly USA	University students	Substance use–alcohol	S26	Moderate
O'Driscoll et al. (81)	The effects of mindfulness-based interventions for health and social care undergraduate students–A systematic review of the literature	-	Undergraduate students	Mental Health/Wellbeing	S11	Critically low
Roy et al. (82)	Food environment interventions to improve the dietary behavior of young adults in tertiary education settings: A systematic literature review	International	Tertiary education	Diet and nutrition	S15/M0	Low
Samson and Tanner-Smith (83)	Single-session alcohol interventions for heavy drinking college students: A systematic review and meta-analysis	Mainly USA	College students	Substance use–alcohol	S73	Critically low
Scott-Sheldon et al. (84)	Efficacy of alcohol interventions for first-year college students: A meta-analytic review of randomized controlled trials	Mainly USA	College students	Substance use–alcohol	M41	Critically low
Scott-Sheldon et al. (85)	Alcohol interventions for college students in greek letter organizations: A systematic review and meta-analysis, 1987 to 2014	USA	College students	Substance use–alcohol	M15	Critically low
Stillwell et al. (86)	Interventions to reduce perceived stress among graduate students: A systematic review with implications for evidence-based practice	USA	Graduate students	Mental Health/Wellbeing	S8	Critically low
Stunden et al. (87)	Tools to reduce first year nursing students' anxiety levels prior to undergoing objective structured clinical assessment (OSCA) and how this impacts on the student's experience of their first clinical placement	International	Nursing students	Mental Health/Wellbeing	S8	Critically low
Turner and McCarthy (88)	Stress and anxiety among nursing students: A review of intervention strategies in literature between 2009 and 2015	US; Canada; UK	Nursing students	Mental Health/Wellbeing	S26	Critically low
Wasson et al. (89)	Association between learning environment interventions and medical student well-being: A systematic review	USA	Medical students	Mental Health/Wellbeing	S28	Low
Webster et al. (90)	A systematic review of the health benefits of Tai Chi for students in higher education	Mainly China	Tertiary or higher education	Physical health	S76	Critically low
Yamaguchi et al. (91)	Effects of short-term interventions to reduce mental health-related stigma in university or college students: A systematic review	International	University students	Mental Health/Wellbeing	S35	Low

*M, Meta-analysis; QA, quality assessment for interventional reviews; S, systematic review*.

**Figure 3:**
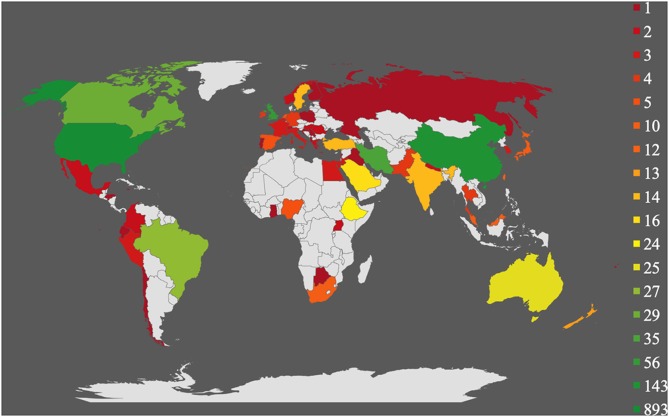
Visual impression of the worldwide distribution of the included original articles.

As shown in Table 1, the included original studies among the eligible review articles were performed in various countries. However, some of the included review articles concentrated exclusively on students' health in particular countries. Figure 3 gives a visual impression of the worldwide spreading of the included original articles showing a strong research focus in USA, UK, and China and a low scientific research interest, for example, in other European countries like Portugal, Poland, Germany, and Italy.

### Substance Use

A total of 36 review articles, comprising 1,312 original articles including ~1,269,602 students, focused on substance use in students. This category was subdivided into the subcategories alcohol (*n* = 18), drugs (licit and illicit; *n* = 9), smoking (*n* = 6), alcohol and drugs (*n* = 2), and smoking and drugs (*n* = 1). These categories, the research question, and the main outcomes of the review articles are sorted by epidemiological review articles and interventional review articles in Table 2. Alcohol consumption was of primary interest in four epidemiological review articles, whereas 14 review articles investigated the effectiveness of strategies to reduce alcohol use in the student collective. The large number of published review articles concerning alcohol consumption shows that especially drinking is a big issue in the student collective. Both, face-to-face programs and internet-based approaches show promising results in reducing drinking behavior. However, few review articles show limited effects and research for long-term impact is lacking. Nine review articles assessed the prevalence of the use of drugs. Further, motives for drug misuse and the role of demographic and psychosocial circumstances are of importance. No interventional review articles met the inclusion criteria. Smoking was of primary interest in six review articles. Five review articles evaluated the smoking behavior among students and one SR/MA focused on the success of anti-smoking policy approaches. In three review articles, a combination of substances [alcohol and drugs (*n* = 2) and smoking and drugs (*n* = 1)] was the object of investigation.

**Table 2:** Main outcomes of the articles included in the systematic umbrella review focusing on “substance use” (*n* = 36).

**References**	**N (students)**	**Research question**	**Main outcome**
**ALCOHOL** ***(n18)***			
**Epidemiological SR/MA (n4)**			
Davoren et al. (12)	23,444 (unclear in 2 studies)	Summary of the current research on alcohol consumption among university students in the Republic of Ireland and the United Kingdom	- Almost two thirds of students reported a hazardous alcohol consumption score - Over 20 % reported alcohol problems over their lifetime using CAGE - Over 20 % exceed sensible limits each week - Narrowing of the gender gap throughout the past decade
Karam et al. (3)	24,645 (unclear in 2 studies)	Presentation of articles on alcohol use among college students in Africa, Asia, Australasia, Europe and South America	- Elevated risk for heavy drinking, with serious immediate health risks, such as drink-driving and other substance use - Longer-term risks, such as alcohol dependence - The prevalence of hazardous drinking in Australasia, Europe and South America appears similar to that in North America, but is lower in Africa and Asia
Newman et al. (41)	97,318	Development of an estimate of self-reported last 30 day alcohol use by students in China	- Estimation of undergraduate student drinking rates in the last 30 days - 66.8 % for male university students and 31.7 % for female university students
Schry and White (45)	24,192	Examination of the relationship between social anxiety and alcohol variables in college students	- Social anxiety was negatively correlated with alcohol use variables - Sig. positively correlated with alcohol-related problems, coping, conformity, and social motives for alcohol use, and positive and negative alcohol outcome expectancies
**Interventional SR/MA (n14)**			
Appiah-Brempong et al. (52)	1,896 (unclear in 2 studies)	Effectiveness of Motivational Interviewing (MI) interventions in reducing alcohol consumption among college students, as compared to no intervention or alternative interventions	- MI interventions were found to be effective in reducing alcohol consumption among college students, when compared to alternative interventions or no intervention
Berman et al. (53)	4,514	Evaluation of mobile intervention efficacy targeting hazardous alcohol use in university students in comparison to controls	- For smartphone apps, one study reported positive results on secondary outcomes - Other showed no differences in comparison to controls for a web-based app and negative results for a native app
Bhochhibhoya et al. (54)	13,141	Evaluation of Internet-based interventions targeting binge drinking among the college population	- Internet-based interventions more effective than traditional print-based interventions - Face-to-face interventions were typically more effective - The Internet as a brief intervention approach can effectively support efforts
Carey et al. (56)	13,750	Evaluation of alcohol abuse prevention interventions for college drinkers	- Participants in risk reduction interventions drank significantly less relative to controls - Students receiving interventions reported fewer alcohol-related problems - Face-to-face interventions predict greater reductions in alcohol-related problems
Carey et al. (57)	28,621	Evaluation of the efficacy of computer-delivered interventions (CDI) to reduce alcohol use among college students	- CDIs reduce the quantity and frequency of drinking among college students - CDIs are generally equivalent to alternative alcohol-related comparison interventions - Small-to-medium within-group effect sizes can be expected for CDIs at short- and long-term follow-ups
Carey et al. (58)	37,480	Determination of the relative efficacy of Computer-delivered interventions (CDIs) and face-to-face interventions (FTFIs) and testing of predictors for intervention efficacy	- Compared to controls, CDI participants reported lower quantities, frequency, and peak intoxication at short-term follow-up, but these effects were not maintained - Direct comparisons between FTFI and CDIs were infrequent, but these trials favored the FTFIs on both quantity and problems measures
Carey et al. (59)	8,621	Evaluation of the efficacy of disciplinary sanctions to prevent future alcohol misuse	- Providing mandated interventions is an effective short-term risk reduction strategy
Dotson et al. (65)	2,050	Investigation of the degree to which computer-delivered stand-alone personalized normative feedback interventions reduce alcohol consumption and alcohol-related harms among college students	- Computer-delivered Personalized Normative Feedback (PNF) is an effective stand-alone approach for reducing college student drinking - Has a small impact on alcohol-related harms - Effects are small but clinically relevant when considered from a public health perspective
Foxcroft et al. (66)	44,958	Determination whether social norms interventions reduce alcohol-related negative consequences, alcohol misuse or alcohol consumption when compared with a control	- No substantive meaningful benefits are associated with social norms interventions for prevention of alcohol misuse - some significant effects were found, the effect sizes are too small, to be of relevance for policy or practice
Ickes et al. (70)	26,356 (unclear in 2 studies)	Determination of the efficacy of alcohol-abuse interventions for college students	- Results indicate interventions found success with decreased drinking, reduction in alcohol problems or consequences, and decreased peer perception of alcohol use
Moreira et al. (80)	7,275	Determination whether social norms feedback reduces alcohol misuse in university or college students	- Web feedback (WF) and individual face-to-face feedback (IFF) are probably effective - No direct comparisons of WF against IFF were found, but WF impacted across a broader set of outcomes and is less costly - Significant effects were more apparent for short-term outcomes - For mailed and group feedback, and social norms marketing campaigns, the results are on the whole not significant and therefore cannot be recommended
Samson and Tanner-Smith (83)	Not provided	Summary of the effectiveness of brief, single-session interventions to reduce alcohol use among heavy drinking college students	- Single-session brief alcohol interventions significantly reduced alcohol use among heavy drinking college students relative to comparison conditions
Scott-Sheldon et al. (84)	24,294	Evaluation of the efficacy of interventions to prevent alcohol misuse by first-year college students	- Behavioral interventions reduce alcohol consumption and alcohol-related problems - Interventions that include personalized feedback, moderation strategies, expectancy challenge, identification of risky situations, and goal setting optimize efficacy
Scott-Sheldon et al. (85)	6,026	Examination of the efficacy of interventions to reduce alcohol consumption and related problems among college student members of Greek letter organizations	- Extant alcohol interventions show limited efficacy in reducing consumption and problems among fraternity and sorority members
**DRUGS** ***(n9)***			
**Epidemiological SR/MA (n9)**			
Bavarian et al. (18)	Not provided	Assessment of prevalence, elucidation ofthe behavior's multietiological nature, and discussion of prevention implications	- The prevalence of illicit use of prescription stimulants (IUPS) varies across campuses - Findings suggest the behavior is multifaceted, as correlates were observed within each stream of influence and level of causation specified by the theory of triadic influence - IUPS is prevalent in, but varies across, colleges and is influenced by intrapersonal and broader social and societal factors
Bennett and Holloway (19)	40,657	Summary on the prevalence of motives for prescription drug misuse (PDM) among university students	- Most prevalent motives for PDM cover some kind of personal enhancement to the user in terms of performance, mental health, or physical health - Fewer than half of users said that they were involved in PDM for pleasure purposes
Benson et al. (20)	89,131	Summary of the current research on rates and demographic and psychosocial correlates of stimulant medication misuse among college students	- Rate of stimulant medication misuse was estimated at 17 % - Review of the literature also revealed that Greek organization membership, academic performance, and other substance use were associated with misuse - Students are misusing primarily for academic reasons, and the most common source for obtaining stimulant medication is peers with prescriptions
Blavos et al. (21)	35,823	Evaluation of the literature on the associated effects of marijuana use on U.S. college students' academic success, including conduct/ legal issues, negative outcomes, normative perceptions, and physical/mental health	- Overall, studies lacked scientific rigor - Researchers relied on convenience samples, used small sample sizes, did not report response rates, or did not report the psychometrics of the instrument - Majority of the studies were conducted at single institutions, limiting external validity
Candido et al. (13)	12,593	Summary of the literature on the use of drugs, licit or not, in Brazilian medical students	- Alcohol and tobacco were the most frequently used licit drugs - The most consumed illicit drugs: marijuana, solvents, “lança-perfume”, and anxiolytics - The male gender showed a tendency of consuming more significant amounts of all kind of drugs, with exception of tranquilizers - Increasing prevalence of drug consumption as the program progressed - Students who do not use psychoactive drugs are more likely to live with their parents, to disapprove drugs consumption, to practice religious beliefs and to be employed
Finger et al. (28)	Not provided	Investigation of the effects of methylphenidate on cognitive enhancement, memory, and performance in medical students	- The prevalence of use reached 16%, with no gender difference - Most students began using the drug after entering the university - The reasons cited to justify it are related to enhancing academic performance
Gebrie et al. (30)	22,351	Estimation of the pooled prevalence of khat chewing and its predictors among Ethiopian university students	- The pooled prevalence of khat chewing was 23.22% - Highest prevalence in Oromia region (31.6%) lowest in Amhara region (18.1%) - Being male, family khat chewing practice, friend khat chewing habit, alcohol drinking and cigarette smoking habit were found to be predictors of khat chewing
Papazisis et al. (42)	19,932	Analysis of the prevalence of lifetime and current use of cannabis among medical students worldwide	- Overall pooled prevalence of lifetime cannabis use was 31.4% - Past-year use was 17.2%, and past-month use was 8.8% - Men displayed higher rates of cannabis use with a pooled relative risk of 1.55
Roncero et al. (43)	88,413	Summary of the literature in the last 25 years about the use of legal and illegal substances by medical students	- Substances used are mainly alcohol (24%), tobacco (17.2%), and cannabis (11.8%) - Use of hypnotic and sedative drugs is common (9.9%) - Rate of use of stimulants is 7.7% and of cocaine, 2.1%; opiate use 0.4% - In some parts of Latin America, up to 14.1% use inhalants - Students in the last years of school have a higher rate of substance use - Use of substances, except for hypnotics and sedatives, is more common among men
**SMOKING** ***(n6)***			
**Epidemiological SR/MA (n5)**			
Bennett et al. (11)	27,921 (unclear in 2 studies)	Evaluation of college-level anti-smoking policies	- Majority (54.5%) of the studies evaluated 100% smoke-free or tobacco-free campus policies - Other types of policies studied included the use of partial smoking restriction and integration of preventive education and/or smoking cessation programs into college-level policies
Cheney et al. (24)	426,348	Examination of studies that included fraternity/ sorority membership in their investigation of smoking behaviors	- Members were more likely to be nondaily smokers - Members who lived in the fraternity/sorority house had higher rates of smoking - Member smoking was associated with alcohol and other substance use
Fevrier et al. (27)	Not provided	Assessment of empirical literature relating to hookah use while focusing on the consequences for regulatory policy	- College students who use hookah are generally not aware of the increased risks for tobacco related diseases as it relates to their behavior - Few public health messages target college-age adults with anti-hookah messages
Guerra et al. (31)	Not provided	Identification of how tobacco use among college students is discussed in the literature	- Many studies published that address the same issue from different perspectives - Studies report differences in consumption of these products with regard to gender, protective factors and those that may predispose the emergence of harmful habits
Haghdoost and Moosazadeh (32)	23,027	Evaluation of the prevalence of smoking in university students in Iran	- Lowest and highest prevalence of smoking among male students was 13.4 and 39.9% - 0.7 and 25.5%, among female students - Smoking frequency among male and female students in Iran's universities is 19.8% and 2.2%
**Interventional SR/MA (n1)**			
Lupton and Townsend (76)	Not provided	Evaluation of university campuses' smoke-free policies	−58.94% of students and 68.39% of faculty supported smoke-free policies - Studies measuring student smoking prevalence indicated a postban reduction (16.5% to 12.8% after 1 year and 9.5% to 7.0% after 3 years) - Only 5% of UK universities were smoke-free compared with 25% of US universities
**ALCOHOL AND DRUGS** (***n2***)			
**Epidemiological SR/MA (n2)**			
Aresi et al. (17)	5,059	Identification of risk and protective factors for alcohol and drug use in students who complete study abroad	- Students increased their alcohol use while abroad and reduced it when they returned home - No evidence of an increase in the negative consequences associated with alcohol misuse - Different pre-departure and abroad factors were related to at-risk behaviors in the host country
Elliott et al. (25)	89,766	Determinations of the effects of family history on substance use and abuse in college and university students	- Family history had a minimal effect on alcohol consumption, with stronger effects on alcohol consequences, alcohol use disorder symptoms and other drug involvement - Students with positive family histories do not drink more, but may be at greater risk for difficulties with alcohol and drugs
**SMOKING AND DRUGS** (*n1)*			
**Interventional SR/MA (n1)**			
Gulliver et al. (69)	Not provided	Summary of technology-based interventions in a tertiary (university/college) setting for tobacco and other drug use (excluding alcohol)	- A range of technology was employed in the interventions, including stand-alone computer programs (*n* = 10), internet (*n* = 5), telephone (*n* = 3), and mobile SMS (*n* = 2) - Although technological interventions have the potential to reduce drug use in tertiary students, very few trials have been conducted, particularly for substances other than tobacco

### Mental Health/Wellbeing

A total of 26 review articles, comprising 762 original articles including ~806,389 students, focused on mental health and wellbeing in students. The research question and the main outcomes of the review articles are sorted by epidemiological review articles and interventional review articles in Table 3. Mental health was of primary interest in nine epidemiological review articles, whereas 17 review articles investigated the effectiveness of strategies to improve the mental health state in the student collective. The topic “stress” in nursing students is primarily studied in the category “mental health.” The identification of stressors, the estimation of prevalence, and the effectiveness of coping strategies to decrease stress or anxiety, were paramount. However, topics like “suicidal thoughts” and “mental health prevention programs” in other student collectives were also evaluated.

**Table 3:** Main outcomes of the articles included in the systematic umbrella review focusing on “mental health/wellbeing” (*n* = 26).

**References**	**N (students)**	**Research question**	**Main outcome**
**Epidemiological SR/MA (n9)**			
Brunsting et al. (23)	85,326	Summary of the strengths and gaps of the literature on undergraduate international student adjustment to U.S. universities	- Acculturative stress, psychological adjustment, social belonging, depression, and anxiety were the most commonly researched outcomes
Elani et al. (9)	7,895	Summary of the available literature on the levels, causes, and impact of stress among dental students	- Dental students experience considerable amounts of stress during their training - Stress is mainly due to the demanding nature of the training - Studies suggest adverse effects of elevated stress on students' health and well-being
Haidar et al. (33)	10,760	Investigation whether stress and anxiety levels encountered during university and college enrolment were associated with higher adiposity or weight changes among students	−11 studies found no association between stress and body mass index or weight change - Five studies did not find a significant association between anxiety and body mass index - Few studies revealed stress and anxiety might be associated with higher or lower weight status
Hurst et al. (34)	Not provided	Investigation of the findings of qualitative research examining stressors in order to understand the major categories of stressors facing college students	- Three themes (relationships, diversity and other) are novel categories of stressors compared with quantitative reviews on the topic
Labrague et al. (36)	1,085 (unclear in 1 study)	Appraisal of existing scientific articles reporting stress perceptions and coping styles in Saudi student nurses	- Moderate to high stress levels, originated mainly from heavy workloads and taking care of patients - When the students' demographic characteristics were taken into account, inconclusive results were found, although some evidence showed higher stress levels in higher level students
McGowan and Murray (38)	1,240	Exploration the concepts of “resilience” and “hardiness” in nursing and midwifery students in educational settings and identification of educational interventions to promote resilience	- Research relating to resilience and resilience education is sparse - There is a weak evidence that resilience and hardiness is associated with slightly improved academic performance and decreased burnout - Studies were heterogeneous in design and limited by poor methodological quality - No study specifically considered student midwives
Mortier et al. (39)	634,662	Estimation of prevalence of suicidal thoughts and behaviors (STB) among college students worldwide	- Estimates of lifetime suicidal ideation, plans, and attempts were 22.3%, 6.1%, and 3.2% - For 12-month prevalence, this was 10.6%, 3.0%, and 1.2%, respectively - Measures of heterogeneity were high for all outcomes, indicating substantial between-study heterogeneity not due to sampling error - Pooled estimates were generally higher for females, as compared with males - Higher STB estimates were also found in samples with lower response rates, when using broad definitions of suicidality, and in samples from Asia.
Sasso et al. (44)	162	Description how dilemmas and environmental, relational, and organizational factors contribute to moral distress in undergraduate student nurses during their clinical experience and professional education	- Inequalities and healthcare disparities, the relationship with the mentor, and students' individual characteristics can all impact negatively on the decisions taken and the nursing care provided, generating moral distress
Thomas and Revell (48)	609 (unclear in 2 studies)	Investigation of the state of knowledge on resilience in nursing students	- Factors that affect resilience were grouped into three themes: support, time, and empowerment - Strategies to promote resilience in nursing students were found in three of the nine articles, but their methods and findings were disparate
**Interventional SR/MA (n17)**			
Alzahem et al. (51)	457 (unclear in 2 studies)	Comparison of the effectiveness of stress management programs in dental education by systematic review of the literature	- Two main strategies have been used to help stressed students - The first strategy includes several components, such as reducing fear of failure and workload pressure due to examinations and requirements - The second strategy includes coping techniques, such as deep breathing exercises - Although positive effects have been reported for most of the programs, these have mainly been evaluated using subjective self-report measures
Bonthuys and Botha (55)	13,257	Determination of the evidence on how the Tomatis® Method, a sound stimulation intervention for improving listening, compares to other self-regulation interventions with tertiary students.	- The Tomatis® Method to be superior to alternative self-regulation approaches in decreasing psychosocial and emotional stressors, as well as enhancing well-being of students - The Tomatis® Method was as effective as alternative approaches in promoting self-awareness and self-monitoring
Conley et al. (61)	9,816	Investigation of the effectiveness of universal mental health prevention programs for higher education students on a range of adjustment outcomes	- Skill-training programs that included a supervised practice component were significantly more effective overall compared to skill-training programs without supervised practice and psychoeducational (information-only) programs - When comparisons on specific outcomes were possible, skill-training programs including supervised practice were significantly more effective than the other two groups of programs in reducing symptoms of depression, anxiety, stress, and general psychological distress, and in improving social-emotional skills, self-perceptions, and academic behaviors and performance
Conley et al. (62)	4,763	Effectiveness of technology-delivered mental health treatment options, such as interventions delivered via computer, smart phone, or other communication or information devices, as preventive interventions for higher education students	- The overall mean effect sizes (ESs) for both universal (0.19) and indicated interventions (0.37) were significant and differed significantly from each other favoring indicated interventions - Skill-training interventions [universal (0.21) and indicated (0.31)], were significant, whereas non-skill-training interventions were only significant among indicated (0.25) programs - For indicated interventions, better outcomes were obtained in those cases in which participants had access to support during the course of the intervention, either in person or through technology (e.g., email, online contact)
Galbraith and Brown (68)	1,900	Identification of the types of interventions that are effective in reducing stress in student nurses	- The most effective interventions provided skills for coping with stressful situations (typically relaxation) and skills for changing maladaptive cognitions - Interventions, which promoted skills to reduce the intensity or number of stressors, were also successful - In most cases, stress interventions did not improve academic performance
Labrague et al. (72)	3,602	Identification of the level of stress and its sources, and exploration of coping methods used by student nurses during nursing education	- Stress levels in nursing students range from moderate to high - Main stressors identified included stress through the caring of patients, assignments and workloads, and negative interactions with staff and faculty - Common coping strategies utilized by nursing students included problem-solving strategies such as developing objectives to resolve problems, adopting various strategies to solve problems, and finding the meaning of stressful events
Labrague et al. (73)	6,591	Appraisal of both quantitative and qualitative studies describing coping strategies utilized by nursing students when faced with stress	- Students utilized problem-focused coping strategies rather than emotion-focused coping strategies - Specific coping behaviors utilized included problem-solving behaviors, self-confident approaches, and seeking of support from family and friends
Li et al. (74)	651	Examination of the efficacy of interventions aimed at improving nursing students' mental health and identification of which form of interventions was effective	- Interventions included psychotherapy, exercise, training program, and others - The results of subgroup analysis showed that depression benefit more from psychotherapy, anxiety benefit from psychotherapy and non-psychotherapy - Interventions were effective in managing stress and systolic blood pressure - Improvements on self-efficacy and diastolic blood pressure was not observed
Lo et al. (75)	2,422	Analysis of interventions to support mental health of health professional students and their effects	- Four comparisons: psychoeducation or cognitive-behavioral interventions compared to alternative education, and mindfulness or relaxation compared to control conditions - Cognitive-behavioral interventions reduced anxiety, depression and stress - Mindfulness strategies reduced stress but not anxiety, depression or burnout - Relaxation strategies reduced anxiety, depression and stress
McCarthy et al. (78)	2,934	Examination of the literature related to the sources of stress, coping mechanisms and interventions to support undergraduate nursing and midwifery students to cope with stress during their undergraduate education	- Students used a variety of coping strategies, both adaptive and maladaptive - These appear to be influenced by their past and present circumstances such as, their needs, what was at stake and their options for coping - Interventions to cope with stress were varied and in the early stages of development - Mindfulness showed some promising positive results
McConville et al. (79)	1,815	Assessment of the effectiveness of mindfulness training in medical and other health professional student population groups and comparison of the effectiveness of the different mindfulness-based programs	- Mindfulness-based interventions decrease stress, anxiety, and depression and improve mindfulness, mood, self-efficacy, and empathy in health profession students - Due to the range of presentation options, mindfulness training can be relatively easily adapted and integrated into health professional training programs
O'Driscoll et al. (81)	1,556	Identification and critical appraisal of the literature on the effects of Mindfulness-Based Interventions for health and social care undergraduate students	- Short-term benefits relating to stress and mood were reported, despite all but one study condensing the curriculum - Gender and personality emerged as factors likely to affect intervention results
Stillwell et al. (86)	373	Evaluation of the existing evidence with the aim of identifying evidence-based self-care interventions for coping with perceived stress	- The interventions varied from a stress management course to mind-body-stress-reduction (MBSR) techniques, such as yoga, breath work, meditation, and mindfulness - All studies measured the outcome of stress with the Perceived Stress Scale - Each study demonstrated a reduction in perceived stress post intervention
Stunden et al. (87)	599	Presentation of the best available evidence into strategies that help reduce first year nursing students' anxiety levels prior to undergoing the Objective Structured Clinical Assessment (OSCA) and clinical placement	- Majority of studies reported simulation session prior to the OSCA increased students confidence and reduced their anxiety levels - This resulted in students' reporting that they valued the OSCA as a worthwhile assessment - There were four major themes: that students were anxious about attending the OSCA; that adequate preparation was seen as a coping strategy; that simulation was a further cause for anxiety; and that the simulation experience could also be used as an OSCA tool
Turner and McCarthy (88)	1,433 (unclear in 3 studies)	Assessment of what progress has been made since 2008, and to examine the strength of current research supporting non-pharmacologic stress management interventions that may be applied to nursing students today	- The majority of interventions aimed to reduce numbers or intensity of stressors through curriculum development or to improve students' coping skills - Some statistically significant support was found for interventions focused on reducing stressors through curriculum development or improving students' coping skills - No statistically significant studies using reappraisal, either alone or in combination with other approaches, were identified
Wasson et al. (89)	8,224	Identification of best practices for undergraduate medical education learning environment interventions that are associated with improved emotional well-being of students	- Studies encompassed a variety of interventions, mental health programs (*n* = 4), mind-body skills programs (*n* = 7), curriculum structure (*n* = 3), multicomponent program reform (*n* = 5), wellness programs (*n* = 4), and advising/mentoring programs (*n* = 3) - Some specific learning environment interventions were associated with improved emotional well-being among medical students - The overall quality of the evidence was low
Yamaguchi et al. (91)	4,257	Identification of the effective approaches to reduce mental health-related stigma in university or college students	- Social contact or video-based social contact interventions seemed to be the most effective in improving attitudes and reducing desire for social distance - Evidence from one study suggests that a lecture that provided treatment information may enhance students' attitudes toward the use of services

### Diet and Nutrition

A total of 6 review articles, comprising 148 original articles including ~50,698 students, focused on diet and nutrition in students. The research question and the main outcomes of the review articles are sorted by epidemiological review articles and interventional review articles in Table 4. Diet and nutrition were of primary interest in two epidemiological review articles whereas four review articles investigated the effectiveness of strategies to improve the dietary intake in the student collective. One of ten medical students is at risk for an eating disorder. Further, students age, color, having children, and being financially independent, are related to higher rates of food insecurity. Various strategies, like in-person interventions, media approaches, and nutrition labeling, are promising in improving the dietary habits among university students.

**Table 4:** Main outcomes of the articles included in the systematic umbrella review focusing on “diet and nutrition” (*n* = 6).

**References**	**N (students)**	**Research question**	**Main outcome**
**Epidemiological SR/MA (n2)**			
Bruening et al. (22)	23,517	Assessment of the prevalence of food insecurity (FI) on postsecondary education institutions, as well as factors related to FI among students and suggested/practiced solutions	- Rates of FI were high among students, with average rates across the gray and peer-reviewed literature of 35% and 42%, respectively - FI was associated with financial independence, poor health, and adverse academic outcomes - The solutions to address food security included those in the intrapersonal, interpersonal, and institutional levels
Jahrami et al. (35)	5,722	Estimation of the prevalence of eating disorders (ED) risk among medical students	- The overall pooled prevalence rate of ED risk was 10.4%, with statistically significant evidence between-study heterogeneity - Prevalence estimates between studies ranged from 2.2 to 29.1%
**Interventional SR/MA (n4)**			
Christoph and An (60)	Not provided	Examination and quantification of the effect of nutrition labels on diet quality in college students	- Sixteen studies found label exposure to be associated with improved diet - Of the 13 studies reporting calories selected or consumed, 8 found that posting labels at the point of purchase decreased calories, 4 found no effect, and 1 found that calories consumed increased after posting labels - Meta-analysis of pre–post studies found a decrease of 36 kcal (P < 0.05) with label exposure
Deliens et al. (63)	15,858	Summary of available literature on interventions aiming to improve dietary intake among university students	- Of the 13 interventions which were effective in improving students' dietary intake, 8 used an intrapersonal approach, with 6 of them using the web or some kind of media to facilitate the intervention - The 5 remaining studies used an environmental approach - Only 1 intervention, using 10 web-based lessons, focused on eating competence and size acceptance to promote healthy eating, was found to be effective in the long-term
Kelly et al. (71)	2,691 (unclear in 2 studies)	Evaluation of nutrition and dietary interventions in college and university settings	- Some in-person interventions show promise in improving students' dietary behaviors - The inclusion of self-regulation components, including self-monitoring and goal setting, may maximize outcomes - Dietary outcomes from online interventions were less promising overall, although they may be more effective with a subset of students early in their readiness to change their eating habits - Environmental approaches may increase the sale of healthy food by serving as visual cues-to-action
Roy et al. (82)	2,910 (unclear in 6 studies)	Evaluation of food environment interventions targeting dietary behavior in young adults in college and university settings	- Information relating to healthy foods through signage and nutrition labels showed improvements in outcomes of interest - Increasing the availability of healthy foods and decreasing the portion size of unhealthy foods improved dietary intake - Price incentives and increased availability of healthy foods combined with nutrition information to increase purchases of healthy foods were identified as having a positive effect on nutrition-related outcomes

### Physical Activity

A total of 4 review articles, comprising 282 original articles including ~220,100 students, focused on the physical activity level in students. The research question and the main outcomes of the review articles are sorted by epidemiological review articles and interventional review articles in Table 5. Physical activity was of primary interest in two epidemiological review articles, whereas two review articles investigated the effectiveness of strategies to influence activity behaviors in the student collective. The length of study is positively associated with an increase in weight and body fat, and the self-reported sedentary behavior or screen time is associated with gender, physical activity behavior, and obesity markers (e.g., BMI and fat percentage). Modifiable factors should be addressed by physical activity promotion approaches with promising personalized interventions.

**Table 5:** Main outcomes of the articles included in the systematic umbrella review focusing on “physical health” (*n* = 4).

**References**	***N* (students)**	**Research question**	**Main outcome**
**Epidemiological SR/MA (n2)**			
Castro et al. (8)	186,630	Identification of the intrapersonal, interpersonal, environmental, and time correlates of sedentary behavior in university students	- Association with sedentary behavior: physical activity (negative association with sitting time), obesity markers (indeterminate associations with TV viewing), and gender - female (null associations with total sitting time and screen time) - Most of the reported correlates of sedentary behavior were intrapersonal, non-modifiable factors
Fedewa et al. (26)	12,831	Assessment of changes in body weight and relative adiposity (%FAT) during college and identify potential moderating variables	- Participants' weight increased 1.55 kg during college, with a 1.17% increase in %FAT - Meta-regression analysis concluded that changes in body weight and % FAT were positively associated with study duration
**Interventional SR/MA (n2)**			
Maselli et al. (77)	11,376	Summary of interventions promoting physical activity (PA) among university students, describing the quality of the evidence, effective strategies, and deficiencies in the interventions employed	- PA promotion interventions should address a range of behavioral determinants - Personalized approaches and PA sessions should be considered in future studies - The high risk of bias of many studies (mainly due to attrition and poor reporting) and missing information about intervention components limit the strength of conclusions about the most effective strategies and the evidence of effectiveness
Webster et al. (90)	9,263	Evaluation of the health benefits of Tai Chi for students in higher education	- Four primary and eight secondary outcomes were found - Tai Chi is likely to benefit participants by increasing flexibility, reducing symptoms of depression, decreasing anxiety, and improving interpersonal sensitivity (primary outcomes) - Secondary outcomes include improved lung capacity, balance, 800/1000m run time, quality of sleep, symptoms of compulsion, somatization and phobia, and decreased hostility

### Sleep

A total of 3 review articles, comprising 107 original articles including ~117,432 students focused on sleep in students. The research question and the main outcomes of the review articles are sorted by epidemiological review articles and interventional review articles in Table 6. Sleep disturbance was of primary interest in one epidemiological review article among Chinese students, whereas two review articles investigated the effectiveness of strategies to improve sleep. There is insufficient evidence on educational approaches for sleep hygiene, whereas cognitive behavioral therapies confirm large effects for improved sleep.

**Table 6:** Main outcomes of the articles included in the systematic umbrella review focusing on “sleep” (*n* = 3).

**References**	***N* (students)**	**Research question**	**Main outcome**
**Epidemiological SR/MA (n1)**			
Li et al. (37)	112,939	This is a meta-analysis of the pooled prevalence of sleep disturbances and its associated factors in Chinese university students	- The overall pooled prevalence of sleep disturbances was 25.7% - The percentages of students dissatisfied with sleep quality and those suffering from insomnia symptoms were 20.3% and 23.6%, respectively - Subgroup analyses revealed that medical students were more vulnerable to sleep disturbances than other student groups - No significant difference between males and females, and across geographic locations
**Interventional SR/MA (n2)**			
Dietrich et al. (64)	1,717	Identification and appraisal of the best available evidence on the effectiveness of sleep education programs in improving sleep hygiene knowledge, sleep hygiene behavior and/or sleep quality vs. traditional strategies	- Insufficient evidence to determine the effectiveness of sleep education on sleep hygiene knowledge, sleep hygiene behavior or sleep quality
Friedrich and Schlarb (67)	2,776	Overview of psychological interventions to improve sleep in college students	- While sleep hygiene interventions provided small to medium effects, the cognitive–behavioral therapy (CBT) showed large effects - CBT approaches provided the best effects for the improvement of different sleep variables in college students

### Media Consumption

A total of 2 review articles, comprising 36 original articles including ~41,896 students, focused on media consumption in students. The research question and the main outcomes of the included systematic reviews and meta-analysis are presented in Table 7. Media consumption was of primary interest in two epidemiological systematic reviews and meta-analysis. The prevalence of internet addiction is high among Chinese students and a crucial issue among medical students.

**Table 7:** Main outcomes of the articles included in the systematic umbrella review focusing on “media consumption” (*n* = 2).

**References**	**N (students)**	**Research question**	**Main outcome**
**Epidemiological SR/MA (n2)**			
Shao et al. (46)	38,245	Estimation of the prevalence of Internet addiction among College Students in the People's Republic of China in order to improve the mental health level of college students and provide evidence for the prevention of Internet addiction	- The pooled Internet addiction detection rate of Chinese college students was 11% - The detection rate was higher in male students (16%) than female students (8%)
Zhang et al. (49)	3,651	Estimation of the prevalence of Internet addiction (IA) among medical students in different countries	- The pooled prevalence of IA among 3,651 medical students is 30.1% - Meta-regression analyses show that the mean age of medical students, gender proportion and the severity of IA are not significant moderators

### Others

A total of 4 review articles, comprising 56 original articles including ~21,612 students focused on “other” topics in students. The research question and the main outcomes of the included systematic reviews and meta-analysis are presented in Table 8.

**Table 8:** Main outcomes of the articles included in the systematic umbrella review focusing on “others” (*n* = 4).

**References**	***N* (students)**	**Research question**	**Main outcome**
**Epidemiological SR/MA (n3)**			
Gambla et al. (29)	8,169	Examination existing reports to determine the comparability of tanning behaviors across multiple U.S. college populations	- High rates of indoor tanning and outdoor tanning were found among college students - Key motivators included appearance, emotion, health perceptions, and the influence of parents, peers, and the media - Misconceptions regarding skin protection, low rates of sun protective behaviors, and tanning dependence were barriers against safe UVR exposure - Understudied demographic factors may account for variance in observed tanning behaviors
Nahar et al. (40)	5,035	Assessment of the skin cancer-related knowledge, attitudes, beliefs, and prevention practices reported in previous studies of medical students	- The attitudes and knowledge of medical students reflect a low level of concern with regard to the perceived importance of skin cancer compared with other forms of cancer despite a high level of concern for the importance of skin cancer prevention - students fail to protect themselves from the sun and have a high interest in tanning bed use
Stellefson et al. (47)	8,408	Summary and critical evaluation of the evidence from existing research on eHealth literacy levels among college students between the ages of 17 and 26 years attending various 4-year colleges and universities located around the world	- All studies measured knowledge and/or behaviors related to college student ability to locate, use, and evaluate eHealth information - many college students lack eHealth literacy skills, suggesting that there is significant room for improvement in college students' ability to obtain and evaluate eHealth information
**Interventional SR/MA (n1)**			
Akinla et al. (50)	Not provided	Description of the outcomes of near-peer mentoring schemes for first-year medical students in the transition phase	- Three outcomes for peer mentoring were identified- professional and personal development, stress reduction, and ease of transitioning - Incidentally, peer mentoring was also found to have facilitated the development of personal and professional attitudes in the mentors

## Discussion

The aim of this study was to provide an overview of review articles on the epidemiology of modifiable health influencing factors and on the evidence of interventions targeting these factors among university students. Thereby, experts as well as stakeholders in the field could gain insights into crucial target points for health promotion and receive guidance about which intervention approaches have shown to be effective and hence, are advisable to implement in practice.

An almost equal amount of review articles with focus on epidemiology and intervention was found. Topics included in the different review articles ranged from physical activity over mental health, substance use, sleep, diet and nutrition, and media consumption. Most frequently targeted was the topic of substance use, particularly alcohol consumption. Also, in the field of mental health, many studies have been conducted—numerous of them dealing with stress. The fields of media consumption, sleep, nutrition, and physical (in)activity are still understudied and more attention needs to be paid to these factors.

For alcohol use and mental health, more intervention studies as compared to epidemiology studies exist. Conversely, in the remaining categories (sleep, diet and nutrition, physical activity, media consumption) the number of epidemiology and intervention studies is not as discrepant. This might be due to the fact, that the overall number of review articles in the area of mental health and alcohol use is higher than in the other categories. Studies that intervene on the setting/environment level as opposed to the individual level are underrepresented. A reason for this might be that environmental strategies could be more difficult to implement and evaluate (92). This is a finding that calls for action since already in the Okanagan Charter the need for a setting-based approach was highlighted (2).

The results gave insights into what interventions seem to be successful. For instance, promising results of interventions in order to reduce drinking behavior could be found in face-to-face programs and internet-based approaches. However, there is a need for future research that can identify approaches with long-term effects. In the field of improving dietary habits of university students in-person interventions, media approaches and nutrition labeling seem to be good strategies. In the area of physical activity promotion interventions future studies should consider personalized interventions. Yet, it is difficult to make conclusions about many of the interventions due to biased studies. In order to improve sleep among university students behavioral cognitive therapy showed larger effects compared to sleep hygiene interventions.

This umbrella review points out a focus of the identified review articles on specific groups like medical or nursing students in the current research landscape that are disproportionately often assessed. This might be explained by the fact that these groups are highly vulnerable, for instance, in terms of mental health problems, as was shown in previous studies (93–96). Future research should aim to incorporate diverse study disciplines and not only target specific groups. Furthermore, the review articles are predominantly conducted in the US, China, and UK. Studies from European countries, like Germany, are underrepresented (97). A large amount of studies included international studies. Yet, the majority of studies still was conducted in the US and UK. It is possible, however, that a search in different languages would have resulted in more studies. Comparisons across different countries and cultures are limited due to differences in school systems. Similarly, the transfer of results and recommendations to other countries needs to be considered with caution as findings might not be generalizable or appropriate for other cultures (98). Therefore, other countries need to take up research in order to identify similarities or differences between countries/cultures.

The quality assessment revealed a low quality for most studies. Therefore, conclusions based on the results need to be drawn carefully and should be investigated in more detail to maintain confidence in the findings. This demonstrates a critical finding and the need for further studies to improve their methodology by adhering to guidelines of how to perform review articles ideally. However, it must be stated that the AMSTAR tool seems very strict. The use of the tool itself is challenging as it requires some experience in order to rate the quality of other studies.

A possible limitation of this umbrella review is that it does not describe the current state of research sufficiently, since there might have been single studies published by now that have not been included into review articles, yet. Furthermore, this umbrella review combined review articles with very different methodologies, which makes it more difficult to compare and interpret results. This point is particularly critical for intervention studies. Another aspect to consider is that only studies in English and German language were included. Moreover, gray literature was not included (99).

The current umbrella review only includes review articles with data of epidemiological and interventional studies on student level. This might seem to contradict the setting-based approach of including everyone in interventions, such as staff/faculty members. This decision had to be made, however, in order to make studies more comparable to each other. Practical implications for health promotion at universities also need to consider research findings incorporating interventions for different groups beyond students as stated, for instance, in the SR by Fernandez et al. (92).

## Conclusion

This umbrella review provides a large overview of the research landscape with regard to modifiable health influencing factors and according interventions. Counting to the methodological strengths is the extensive amount of studies reviewed in duplicate as part of the general conduct according to the PRISMA guidelines (15). Further making this umbrella review exceptional is that it combines a wide spectrum of health topics that were displayed in the different categories: from physical activity to diet and nutrition, mental health, substance use and media consumption, a diverse set of topics is covered. In addition, a salutogenic approach was focused. This umbrella review is not disease oriented but rather oriented toward positive health and modifiable factors (health determinants and health behaviors). It provides a great overview for those who quickly need to gain information about the current evidence of modifiable health influencing factors in the context of health promotion among university students.

## Author Contributions

All authors contributed to the conception, analysis, and interpretation of the manuscript. All authors read and approved the final document.

## Conflict of Interest

The authors declare that the research was conducted in the absence of any commercial or financial relationships that could be construed as a potential conflict of interest.
